# Reelin Affects Signaling Pathways of a Group of Inhibitory Neurons and the Development of Inhibitory Synapses in Primary Neurons

**DOI:** 10.3390/ijms22147510

**Published:** 2021-07-13

**Authors:** Seong-Eun Lee, Gum Hwa Lee

**Affiliations:** College of Pharmacy, Chosun University, 309 Pilmun-daero, Dong-gu, Gwangju 61452, Korea; leessung33@naver.com

**Keywords:** reelin, inhibitory synapse, GABAergic neurons, Akt phosphorylation

## Abstract

Reelin is a secretory protein involved in a variety of processes in forebrain development and function, including neuronal migration, dendrite growth, spine formation, and synaptic plasticity. Most of the function of Reelin is focused on excitatory neurons; however, little is known about its effects on inhibitory neurons and inhibitory synapses. In this study, we investigated the phosphatidylinositol 3-kinase/Akt pathway of Reelin in primary cortical and hippocampal neurons. Individual neurons were visualized using immunofluorescence to distinguish inhibitory neurons from excitatory neurons. Reelin-rich protein supplementation significantly induced the phosphorylation of Akt and ribosomal S6 protein in excitatory neurons, but not in most inhibitory neurons. In somatostatin-expressing inhibitory neurons, one of major subtypes of inhibitory neurons, Reelin-rich protein supplementation induced the phosphorylation of S6. Subsequently, we investigated whether or not Reelin-rich protein supplementation affected dendrite development in cultured inhibitory neurons. Reelin-rich protein supplementation did not change the total length of dendrites in inhibitory neurons in vitro. Finally, we examined the development of inhibitory synapses in primary hippocampal neurons and found that Reelin-rich protein supplementation significantly reduced the density of gephyrin–VGAT-positive clusters in the dendritic regions without changing the expression levels of several inhibitory synapse-related proteins. These findings indicate a new role for Reelin in specific groups of inhibitory neurons and the development of inhibitory synapses, which may contribute to the underlying cellular mechanisms of *RELN*-associated neurological disorders.

## 1. Introduction

Reelin is a large glycoprotein that is secreted by Cajal-Retzius cells in the marginal zone during cortex formation in the fetal brain and is then released from a group of inhibitory neurons after birth [1,2]. The Reelin gene (*Reln*) was first discovered as a regulator of neuronal migration during neocortex formation [1,3]. Further studies have elucidated its role in synaptic plasticity, as well as dendrite development and spine formation for excitatory neurotransmission [4,5,6,7]; thus, *RELN* is a candidate gene for several psychiatric disorders, such as schizophrenia, major depression, and autism spectrum disorder [3,8,9,10]. Intracellular signaling pathways activated by Reelin have been investigated in accordance with its various roles. Reelin binds to apolipoprotein E receptor 2 (ApoER2) and very low-density lipoprotein receptor (VLDLR) to activate C3G/Rap1 for neuronal migration and phosphoinositide 3-kinase (PI3K)/Akt/mTOR for dendrite and spine development [3]. In addition, Reelin has been reported to be involved in synaptic plasticity through the activation of MAPK/ERK1/2 via an unknown receptor [4,5,11]. While most of Reelin’s functions focus on excitatory neurons and excitatory synapses, little is known about its effects on inhibitory neurons.

γ-Aminobutyric acid (GABA) is released by GABAergic neurons in inhibitory synapses to provide inhibitory neurotransmission to neural circuits in the brain. Inhibitory neurotransmission reduces synaptic activation and modulates the frequency of action potential firing in postsynaptic neurons, which is essential in balancing excitation and inhibition when processing neural information [12]. Since inhibitory synapses in GABAergic circuits function as platforms for neurotransmission, morphological and compositional alterations in theses synapses are associated with several neurological and neuropsychiatric diseases, such as autism spectrum disorder, schizophrenia, and epilepsy [13,14,15,16,17,18].

Previous studies have shown that alterations in neuronal positioning of inhibitory neurons in *Reln*-deficient mice were apparently due to impaired migration of excitatory neurons in the neocortex, and that inhibitory neuron-specific deletion of *Reln*, which disrupts Reelin supply in the postnatal period, did not cause any changes in behavior, including learning and memory [19,20]; however, given that inhibitory neurons are comprised of diverse subgroups, each with different characteristics, it necessary to study the effects of Reelin on the cellular function of specific inhibitory neurons. Additionally, the development of inhibitory synapses is regulated by several factors, such as N-methyl-D-aspartate receptor or GABA_A_ receptor activity, the expression of pre- or postsynaptic molecules, and brain-derived neurotrophic factor (BDNF) [21,22]. It is interesting to examine the effects of Reelin on inhibitory synapse formation in principal neurons.

In this study, we investigated the effects of Reelin on (1) the phosphorylation of Akt and ribosomal S6 protein in individual inhibitory neurons using an immunofluorescence assay and (2) dendrite outgrowth of inhibitory neurons. To study the development of inhibitory synapses, we analyzed the intensity and density of gephyrin- and vesicular GABA transporter (VGAT)-positive clusters in defined dendritic regions of primary hippocampal neurons. Additionally, we examined the expression of proteins or mRNAs that are related to inhibitory synapses under Reelin-supplemented conditions.

## 2. Materials and Methods

### 2.1. Reagents and Antibodies

The primary antibodies used in this study were rabbit anti-phospho-S6 235/236 (1:3000 for Western blotting, 1:1000 for immunofluorescence, Cell Signaling, Danvers, MA, USA, #4858, #2211), rabbit anti-phospho-S6 240/244 (1:5000 for Western blotting, 1:1000 for immunofluorescence, Cell Signaling, Danvers, MA, USA, #5364), rabbit anti-phospho-Akt (1:5000 for Western blotting, 1:1000 for immunofluorescence, Cell Signaling, Danvers, MA, USA, #4060), rabbit anti-Akt (1:5000, Cell signaling, Danvers, MA, USA, #4691), chicken anti-Map2 (1:30,000, Abcam, Cambridge, UK, ab5392), mouse anti-gephyrin (1:1000, Synaptic systems, Goettingen, Germany, 147-021), mouse anti-gephyrin (1:3000, Synaptic systems, Goettingen, Germany, 147-111), rabbit anti-VGAT (1:3000 for Western blotting, 1:1000 for immunofluorescence, Synaptic Systems, Goettingen, Germany, 131 002), mouse anti-GAD67 (1:3000 for Western blotting, 1:1000 for immunofluorescence Millipore, Temecula, CA, USA, MAB5406), anti-GAD65 (1:3000, Abcam, Cambridge, UK, ab26113), somatostatin (1:100, Santacruz, Oregon, USA, sc-55565), and mouse anti- β-actin-HRP (1:50,000, Sigma, St. Louis, MO, USA, A3854). The secondary antibodies were HRP-conjugated anti-rabbit and anti-mouse (Invitrogen, Rockford, IL, USA, G21040, G21234) antibodies conjugated to Alexa Fluor 488 goat anti-chicken (Invitrogen, A11039), Alexa Fluor 555 goat anti-mouse (Invitrogen, Eugene, OR, USA, A21422), and Alexa Fluor 647 goat anti-rabbit (Invitrogen, Eugene, OR, USA, A21244).

### 2.2. Primary Neuronal Culture

The cerebral cortices and hippocampi were dissected from the brain of ICR mouse (embryonic day 15.5) and digested with 0.25% trypsin (Gibco, Grand Island, NY, USA)–DNase I (Sigma-Aldrich, St. Louis, MO, USA) at 37 °C for 20 min; trypsin was inactivated using fetal bovine serum (Invitrogen, Carlsbad, CA, USA). The dissociated neurons were placed on poly-L-lysine (Sigma, St. Louis, MO, USA, P5899) precoated plates and cultured in neurobasal medium supplemented with 2% B27 supplement, Glutamax, and penicillin–streptomycin (Gibco, Grand Island, NY, USA). The cells were cultured at 37 °C in a saturated atmosphere containing 95% air and 5% CO_2_, and the medium was replaced with fresh medium a day after isolation. Afterward, half of the medium was replaced every 3–4 days. All animals used in this study were handled in accordance with protocols approved by the Institutional Animal Care and Use Committee at Chosun University. Because culture hippocampal neurons are more homogenous in their morphology and biophysical properties compared to cortical neurons [23,24,25,26,27,28], we used hippocampal neurons to examine dendrite growth and inhibitory synapse formation on excitatory neurons to reduce experimental variability.

### 2.3. Production of Recombinant Reelin and Semi-Purification

HEK293T cells were cultured in Dulbecco’s modified Eagle’s medium (Hyclone, Logan, UT, USA, SH30243.01) with 10% fetal bovine serum and transfected with mock (control) or *Reln* cDNA construct pCrl, kindly provided by G. D’Arcangelo [1], using Transit (Mirus, Madison, WI, USA, 61044894). The medium of the transfected cells was replaced with serum-free medium at 24 h after transfection and collected for 2 days. The supernatants were centrifuged for the removal of cell debris and concentrated using Amicon Ultra-15 filters (Merck, Carrigtwohill, Ireland, UFC903008) at 2680× *g* for 20 min prior to addition to neuronal cultures as described previously [6]. Primary neurons were replenished with Reelin-containing concentrates, referred to as Reelin-rich protein supplementation.

### 2.4. RNA Isolation and RT-PCR Analysis

Total RNA was obtained from primary neurons using a GeneJET RNA Purification Kit (Thermo Scientific, Vilnius Lithuania, K0732) according to the manufacturer’s instructions. The total RNA (0.5–1 μg) was reverse-transcribed to generate cDNA using the iScript^TM^ cDNA Synthesis Kit (bio-rad, Hercules, CA, USA, 1708891). The mRNA quantification was performed using a StepOne real-time PCR system (Applied Biosystems) and SYBR Green PCR Master Mix (Applied Biosystems, Warrington, UK, 4309155). Transcript-specific primers used in the quantitative RT-PCR were: Gad67, F: 5′-CACAGGTCACCCTCGATTTTT-3′, R: 5′-ACCATCCAACGATCTCTCTCATC-3′, PrimerBank ID: 31982847a1; Gad65, F: 5′-TCCGGCTTTTGGTCCTTC-3′, R: 5′-ATGCCGCCCGTGAACTTTT-3′, PrimerBank ID: 6679925a1; Neuroligin1, F: 5′-GGTACTTGGCTTCTTGAGCAC-3′, R: 5′-AAACACAGTGATTCGCAAGGG-3′, PrimerBank ID: 28972598a1; Neuroligin2, 5′-TGTCATGCTCAGCGCAGTAG-3′, 5′-GGTTTCAAGCCTATGTGCAGAT-3′, PrimerBank ID: 33989614a1; Semaphorin 4D, 5′-CCTGGTGGTAGTGTTGAGAAC-3′, 5′-GCAAGGCCGAGTAGTTAAAGAT-3′, PrimerBank ID: 7305471a1; glyceraldehyde 3-phosphate dehydrogenase (Gapdh), F: 5′-AGGTCGGTGTGAACGGATTTG-3′, R: 5′-TGTAGACCATGTAGTTGAGGTCA-3′, PrimerBank ID: 9055212a1F. GAPDH was used as a reference gene for normalization. The relative quantification of the mRNA was calculated using the Pfaff1 method [29].

### 2.5. Western Blot Analysis

Primary cortical neurons were lysed at designated times with radioimmunoprecipitation assay (RIPA) buffer. The protein extracts were separated using 6% or 8% SDS-PAGE gels and transferred to a 0.22-µm nitrocellulose (NC) membrane. The membrane was incubated in 5% skim milk blocking buffer for 1 h at room temperature and then with a primary antibody at 4 °C overnight. After washing with Tris-buffered saline and Tween 20 (TBST), a secondary antibody was supplemented to the membrane for 1 h at room temperature. Membranes were developed with Western blot ECL solution (Neutronex, Goryeong, South Korea, NXECL-2011). Total Akt, Erk, or β-actin were used as loading controls. 

### 2.6. Immunofluorescence

Primary hippocampal neurons, grown on glass coverslips coated with poly-L-lysine, were fixed in 4% paraformaldehyde (PFA)–phosphate buffered saline (PBS) for 15 min and then permeabilized by 0.1% Triton X-100–PBS for 10 min. After incubation with 10% normal goat serum (NGS) blocking buffer for 1 h at room temperature, the neurons were incubated with primary antibody at 4 °C overnight. After washing with PBS, the neurons on the coverslip were incubated with secondary antibodies for 1 h at room temperature. The neurons were washed with PBS 3 times for 5 min and then were mounted on positively charged glass slides with mounting media (Thermo Scientific, Kalamazoo, MI, USA, 9990402). The neurons were imaged using a Nikon A1 confocal laser microscope system. 

### 2.7. Dendrite Analysis and Quantification of Gephyrin–VGAT-Positive Clusters

The ImageJ program and NeuronJ plugin were used to trace dendrites. The density, area, and intensity of gephyrin or gephyrin–VGAT-positive clusters were measured in primary of secondary dendrites of excitatory hippocampal neurons. The background intensity of each channel was subtracted and the imageJ function Adjust/Threshold was applied to each image under the same conditions to quantify the cluster size. We analyzed the total lengths of the dendrites of 30 to 90 cells in each group, while 30 to 40 dendritic fields were randomly selected from pyramidal neurons in each experiment. All analyses were performed by blinded experimenters with regard to treatment. 

### 2.8. Statistical Analysis

The results are presented as means (standard deviation, SD) and were analyzed using the Mann–Whitney U test or Kruskal–Wallis test followed by Dunn’s comparison test. The results were averaged from multiple experiments (n), which consisted of several wells over 3 different cultures, as indicated in the figure legends. The results were considered statistically significant when the *p* values were less than 0.05.

## 3. Results

### 3.1. Reelin Does Not Phosphorylate Akt or S6 Protein in Numerous Inhibitory Neurons

**Figure 1:**
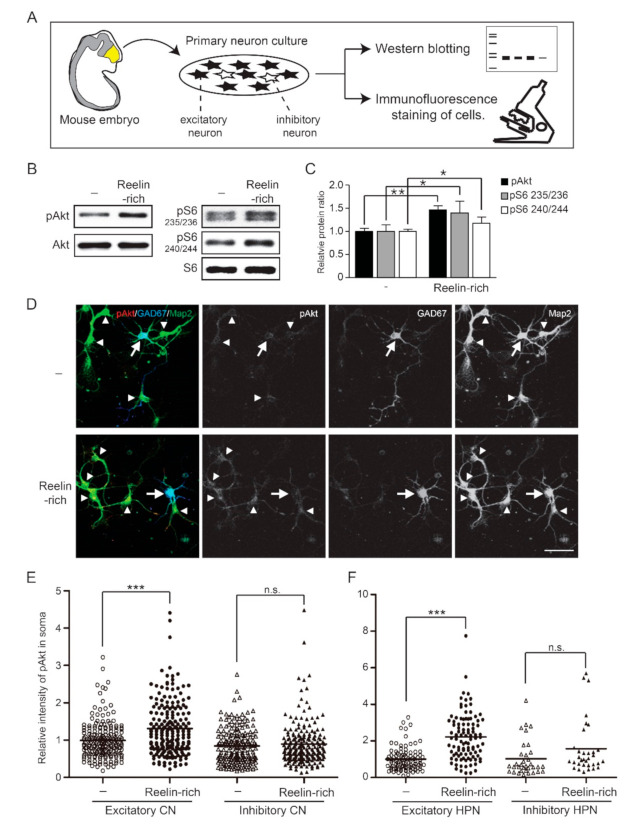
Phosphorylation of Akt and ribosomal S6 protein by Reelin-rich protein supplementation in individual inhibitory neurons: (**A**) Schematic diagram of the neuronal culture and methodology. Primary cortical or hippocampal neurons isolated from the brain of mouse embryos are composed of mostly excitatory neurons and some inhibitory neurons. The protein extract was used for Western blotting, regardless of the cell type, but individual neurons were visualized with a confocal microscope using immunofluorescence staining of cells. (**B**) Five-day in- vitro cortical neurons were supplemented with Reelin-rich protein for 20 min and assayed using Western blotting. The levels of phosphorylated Akt and S6 were significantly increased by Reelin-rich protein supplementation. (**C**) The results were averaged from three individual experiments and analyzed using the Mann–Whitney U test. (**D**) Five-day in vitro cortical neurons were supplemented with Reelin-rich protein for 20 min and imaged by confocal microscopy using Map2 (neuron marker), GAD67 (inhibitory neuron marker), or phospho-Akt antibodies. GAD67-positive inhibitory neurons are indicated with arrows and GAD67-negative excitatory neurons with arrowheads. A significant increase in the level of phosphorylated Akt was shown in GAD67-negative–Map2-positive excitatory neurons, but not in GAD67-positive inhibitory neurons. (**E**) The fluorescence intensity of the soma was quantified and the sample distribution of individual cortical neurons is shown (number of excitatory neurons = 200, number of inhibitory neurons = 200). (**F**) Fluorescence intensity quantification and sample distribution of individual hippocampal neurons (number of excitatory neurons = 100, number of inhibitory neurons = 33–34). The results were analyzed using the Kruskal–Wallis test followed by Dunn’s comparison test. Note: * *p* < 0.05, ** *p* < 0.01, *** *p* < 0.001; n.s., nonsignificant. Scale bar, 50 μm.

**Figure 2:**
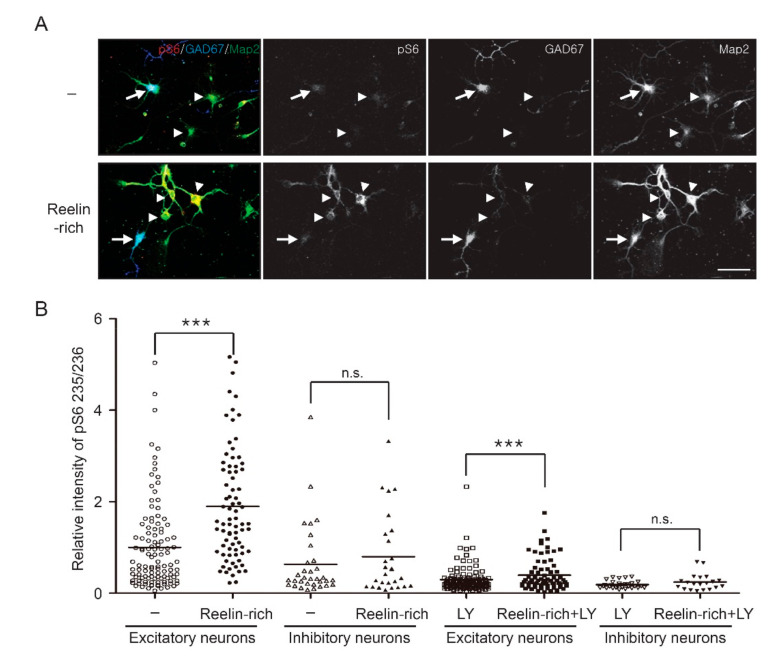
Phosphorylation status of ribosomal S6 protein, a downstream molecule of the PI3K/Akt pathway, by Reelin-rich protein supplementation in individual inhibitory neurons: (**A**) Five-day in vitro hippocampal neurons were supplemented with Reelin-rich protein for 20 min and imaged using confocal microscopy using Map2, GAD67, or phospho-S6 (serine 235/236) antibodies. GAD67-positive inhibitory neurons are indicated with arrows and GAD67-negative excitatory neurons with arrowheads. (**B**) The fluorescence intensity of the soma was quantified and the sample distribution of individual neurons is shown (number of excitatory neurons = 75–119, number of inhibitory neurons = 20–33). A significant increase in the levels of phosphorylated S6 was shown in GAD67-negative–Map2-positive excitatory neurons, but not in GAD67-positive inhibitory neurons. S6 phosphorylation in both excitatory and inhibitory neurons was completely suppressed under the PI3K inhibitor LY294002 (10 μM), regardless of Reelin-rich protein supplementation. The results were analyzed using the Kruskal–Wallis test followed by Dunn’s comparison test. Note: *** *p* < 0.001; n.s., nonsignificant. Scale bar, 50 μm.

Primary cortical or hippocampal neurons isolated from the brains of mouse embryos are composed of mostly excitatory neurons and some inhibitory neurons (Figure 1A). The protein extract was used for Western blotting, regardless of the cell type, but individual neurons were visualized with a confocal microscope using immunofluorescence staining of cells. Western blot analysis showed that Reelin-rich protein supplementation phosphorylated Akt and a downstream effector, ribosomal S6 protein, within 20 min after the supplementation in 5-day in vitro (DIV) primary cortical neurons (Figure 1B). The relative levels of Akt phosphorylated at serine 473 and of S6 protein phosphorylated at serine 235/236 or serine 240/244 were 1.46 (0.09), 1.40 (0.26), and 1.17 (0.14) compared to control, respectively (controls = 1.00 (0.07), 1.00 (0.14), and 1.00 (0.05), respectively) (Figure 1C). Since inhibitory neurons make up approximately 10–20% of all neurons in primary neuron cultures, the levels of phosphorylated signaling proteins presented by Western blotting do not indicate the distinct response of inhibitory neurons to Reelin. To investigate whether the Reelin signaling pathway is stimulated in individual inhibitory neurons, an immunofluorescence assay was performed on primary cortical neurons using antibodies against GAD67, a marker of inhibitory neurons; Map2, a dendritic marker of neurons; and phosphorylated Akt at serine 473. Both GAD67- and Map2-positive neurons were considered inhibitory neurons, while GAD67-negative–Map2-positive neurons were considered excitatory neurons. Interestingly, the intensity of phosphorylated Akt in the soma or dendrites of excitatory neurons significantly increased in response to Reelin-rich protein supplementation, but not in those of inhibitory neurons (Figure 1D,E). The relative intensity levels of phosphorylated Akt in response to Reelin-rich protein supplementation in the soma of excitatory and inhibitory neurons were 1.32 (0.72) and 0.90 (0.57), respectively, compared to the controls (1.00 (0.48) and 0.85 (0.47), respectively). These results were also observed in primary hippocampal neurons (Figure 1F), where the relative intensity level of phosphorylated Akt in response to Reelin-rich protein supplementation was 2.22 (1.27) compared to the controls (1.00 (0.63)) in excitatory neurons and 1.58 (1.47) compared to control (1.02 (0.97)) in inhibitory neurons. In addition, we further investigated whether or not the downstream signaling molecule, ribosomal S6 protein, of the PI3K/Akt pathway was affected by Reelin in inhibitory neurons. In accordance with Akt phosphorylation, Reelin-rich protein supplementation-induced phosphorylation of S6 at serine 235/236 was confirmed in excitatory neurons but not in inhibitory neurons (Figure 2A,B). The phosphorylation levels of S6 in both excitatory and inhibitory neurons were completely suppressed by the PI3K inhibitor LY294002, regardless of Reelin supplementation (Figure 2B); however, the levels of phosphorylated S6 were significantly increased in excitatory neurons in response to Reelin-rich protein supplementation after treatment of PI3K inhibitor, which suggests the involvement of other signaling pathways.

### 3.2. Reelin-Rich Protein Supplementation Induces Phosphorylation of S6 in Somatostatin-Positive Inhibitory Neurons

**Figure 3:**
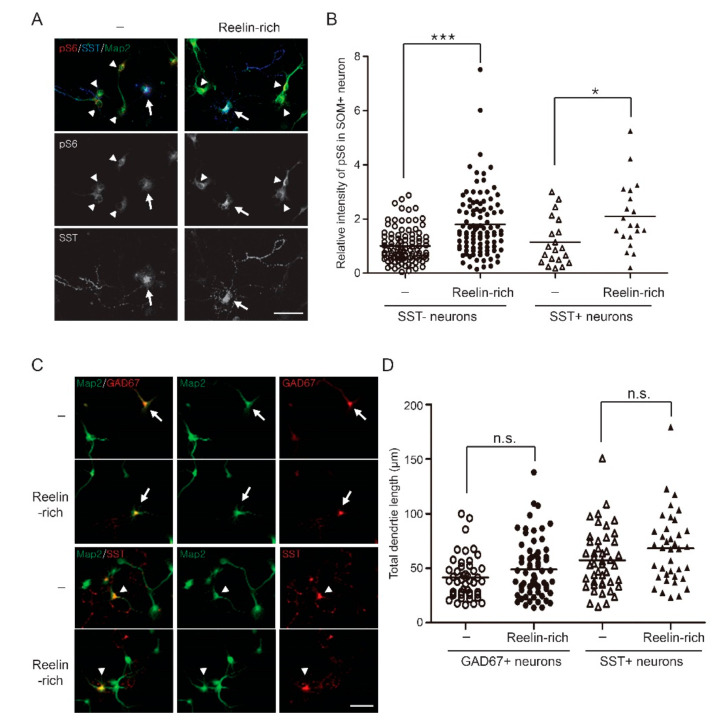
Reelin-rich protein-induced phosphorylation of S6 without dendrite outgrowth effect in SST-positive inhibitory neurons: (**A**) Five-day in vitro hippocampal neurons were supplemented with Reelin-rich protein for 20 min and imaged by confocal microscopy using Map2, GAD67, or phospho-S6 (serine 235/236) antibodies. Somatostatin-positive inhibitory neurons are indicated with arrows and somatostatin-negative excitatory neurons with arrowheads. (**B**) The fluorescence intensity of the soma was quantified and the sample distribution of individual SST-positive inhibitory neurons is shown (number of SST-negative neurons = 99–102, number of SST-positive inhibitory neurons = 20). (**C**) Four-day in vitro hippocampal neurons were supplemented with Reelin-rich protein for 24 h and imaged by confocal microscope using Map2, GAD67, or somatostatin antibodies. GAD67-positive inhibitory neurons are indicated with arrows and somatostatin-positive inhibitory neurons with arrowheads. (**D**) Fluorescence intensity quantification and sample distribution of individual hippocampal neurons (number of GAD67-positive inhibitory neurons = 47–67, number of SST-positive inhibitory neurons = 38–46). No significant differences in dendrite outgrowth were observed between most inhibitory neurons and SST-expressing neurons. The results were analyzed using the Kruskal–Wallis test followed by Dunn’s comparison test. Note: * *p* < 0.05, *** *p* < 0.001; n.s., nonsignificant. Scale bar, 50 μm.

GABAergic inhibitory neurons are heterogenous and can be classified by their morphology, electrophysiological properties, molecular identities, and neurochemical properties. In the classification of neurochemical properties, parvalbumin (PV) and somatostatin (SST)-expressing inhibitory neurons represent two broad and rarely overlapping subtypes in the neocortex and hippocampus [30,31]. Since PV is not sufficiently expressed in developing neurons [32], and because several antibodies used in this study did not detect endogenous parvalbumin in the immunofluorescence assay, we analyzed SST-positive inhibitory neurons to study the subtype-specific response of inhibitory neurons to Reelin. In SST-positive inhibitory neurons, Reelin-rich protein supplementation significantly phosphorylated S6 at serine 235/236 (Figure 3A,B). The relative intensity levels of phosphorylated S6 at serine 235/236 in response to Reelin-rich protein supplementation were 1.80 (1.18) and 2.10 (1.23) in SST-negative and SST-positive neurons, respectively (controls: 1.00 (0.63) and 1.15 (0.88), respectively). Since GAD67-positive inhibitory neurons and SST-expressing interneurons made up approximately 13.5% and 3.3% of all neurons in our culture, respectively, we presumed that Reelin may not activate the PI3K/Akt/S6 signaling pathway in PV-expressing inhibitory neurons, which is the major subtype of inhibitory neurons.

### 3.3. Reelin-Rich Protein Supplementation Does Not Affect Dendrite Outgrowth of Inhibitory Neurons in Primary Cultures

Since dendrite outgrowth of immature neurons is reduced in *Reln*-deficient mice and rescued by Reelin supplementation [7], and because Reelin has a tropic effect on dendrite outgrowth through the PI3K/Akt pathway [33], we studied whether Reelin-rich protein supplementation enhances dendrite outgrowth of inhibitory neurons. We supplemented Reelin-rich protein to 4 DIV hippocampal neurons for 24 h and performed an immunofluorescence assay to measure total dendrite lengths; however, no significant differences in dendrite outgrowth were observed between most inhibitory neurons and SST-expressing neurons in our culture conditions (Figure 3C,D).

### 3.4. Reelin-Rich Protein Supplementation Reduces the Density of Inhibitory Synapses

**Figure 4:**
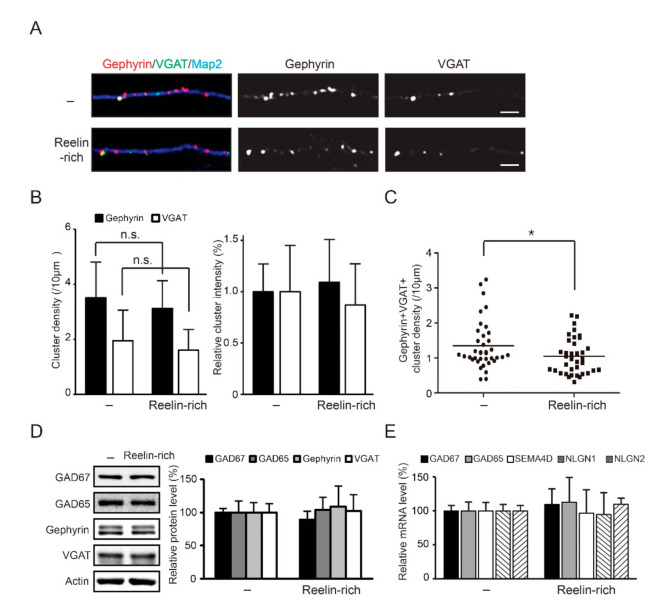
Reduced density of inhibitory synapses by Reelin-rich protein supplementation without changes in expression of inhibitory synapse-related proteins. (**A**) Twelve-day in vitro hippocampal neurons were supplemented with Reelin-rich protein for 12 h and imaged using immunofluorescence followed by confocal microscopy. Gephyrin-positive or VGAT-positive clusters were seen on the dendrites of Map2-positive excitatory neurons. (**B**,**C**) The numbers and fluorescence intensity levels of synaptic gephyrin-positive or VGAT-positive clusters were quantified and the sample distribution of both gephyrin- and VGAT-positive clusters is shown. Each density of gephyrin- or VGAT-positive clusters did not change, but the density of both gephyrin- and VGAT-positive clusters decreased significantly. The results (number of dendritic fields = 37–39) were analyzed using the Mann–Whitney U test. (**D**) Twelve-day in vitro cortical neurons were treated with Reelin-rich protein for 12 h and assayed using Western blot analysis. The expression levels of inhibitory synapse-related proteins were not changed in response to Reelin-rich protein supplementation. The results were averaged from four individual experiments. (**E**) Twelve-day in vitro cortical neurons were treated with Reelin-rich protein for 3 h and mRNA contents were quantified using quantitative real time-PCR. The transcripts levels of inhibitory synapse-related genes were not changed in response to Reelin-rich protein supplementation. The results were analyzed using the Mann–Whitney U test. Note: * *p* < 0.05 and n.s., nonsignificant. Scale bars, 5 μm.

Inhibitory synapses located on the dendrites of pyramidal neurons are important for dendritic integration [34]. To study whether Reelin affects the development of inhibitory synapses, we analyzed inhibitory synapses in 12 DIV hippocampal neurons using immunofluorescence assay and confocal microscopy. We examined dendritic inhibitory synapses in hippocampal excitatory neurons for morphological studies because of their better homogeneity than cortical neurons [23,24,28]. Gephyrin, an inhibitory postsynaptic marker, and VGAT, an inhibitory presynaptic marker, were used to measure the density of inhibitory synapses. The thickness of the primary or secondary dendrites used for analysis was approximately 2μm. After 12 h of Reelin-rich protein supplementation, the densities of gephyrin- or VGAT-positive clusters were unchanged (Figure 4A,B); however, the densities of co-localized gephyrin- and VGAT-positive clusters decreased significantly (Figure 4A,C). The average density of gephyrin–VGAT clusters per 10 μm of dendrite with Reelin-rich protein supplementation was 1.05 (0.52) (control, 1.35 (0.68_). 

Western blot analysis showed that the expression levels of inhibitory synapse-related proteins, including GAD67, GAD65, gephyrin, and VGAT, were unchanged 12 h after Reelin-rich protein supplementation (Figure 4D). In addition, the mRNA levels of genes, including GAD67, GAD65, Semaphorin4D, Neuroligin1, and Neuroligin2, were not altered (Figure 4E). 

These data suggest that Reelin influences the development of inhibitory synapses on excitatory neurons without directly activating the PI3K/Akt/S6 pathway in numerous inhibitory neurons.

## 4. Discussion

Reelin is a critical protein in migration of excitatory neurons during cortex formation; however, it has been reported that it does not directly affect the migration and positioning of inhibitory neurons [19,20]. Since Reelin is involved in several postnatal processes in the development and function of excitatory neurons, such as dendrite outgrowth, spine formation, and synaptic plasticity [5,6,7,33], it is interesting to study Reelin and the functions of inhibitory neurons. Reelin plays several roles through the activation of the adaptor protein Dab1 and the subsequent PI3K/Akt signaling pathway for dendrite and spine development in excitatory neurons [6,7,33]. The canonical Reelin signaling pathway examined using Western blotting does not represent the responses of individual neurons to Reelin. Since inhibitory neurons make up approximately 10–20% of all neurons, the distinct responses of inhibitory neurons to stimuli can be masked by the responses of excitatory neurons in Western blotting. In this study, an immunofluorescence assay was used to examine Reelin-induced activation of intracellular signaling in individual inhibitory neurons. We found that Reelin-rich protein supplementation did not phosphorylate Akt and S6 protein in most inhibitory neurons. Inhibitory neurons are heterogenous and may respond to Reelin in different ways, depending on the subgroups. In this study, SST-expressing interneurons significantly phosphorylated Akt and S6 under Reelin-rich protein-supplemented conditions, similar to excitatory neurons. It is shown for the first time in this work that Reelin has a differential effect on signaling pathways in certain groups of inhibitory neurons. Because studies on subgroup-specific inhibitory neurons during cortex development are rare, neuronal migration and maturation of SST-expressing neurons related with Reelin needs to be investigated further in vivo. In addition, SST-expressing interneurons have been reported to contribute to hippocampal memory by regulating mTORC1, which is part of an upstream signaling complex involving S6 [35]; thus, mTORC1 is an interesting target for studying the roles of Reelin and SST-expressing interneurons in learning and memory. Furthermore, since SST-expressing interneurons made up about a quarter of the inhibitory neurons in our culture conditions, we presume that Reelin may not activate the PI3K/Akt/S6 signaling pathway in PV-expressing interneurons, the major subtype of inhibitory neurons. 

In this study, we used Reelin-rich protein supplementation. Due to difficulties purifying Reelin, Reelin-conditioned medium has been used to examine the function of Reelin in vitro or ex vivo [5,33,36]; however, the conditioned medium might have several limitations, including other secreted proteins, degradation, and differing protein levels compared to the control medium. Previous studies have elucidated the new role of purified full-length Reelin in the activation of intracellular signaling pathways, such as MAPK/ERK1/2 [4,11], which remains unchanged with the Reelin-conditioned medium; therefore, it could be interesting to further investigate the function of Reelin with purification in inhibitory neurons.

Since Dab1 is critical for the activation of the PI3K/Akt/S6 signaling pathway by Reelin, and because its distribution in tissue is restricted [3,37], it is necessary to investigate the expression of Dab1 in inhibitory neurons to elucidate the cellular mechanisms behind its function; however, due to the limited availability of commercial Dab1 antibodies against mouse species for immunocytochemistry, it is not easy to directly determine the cell-specific expression of Dab1 at this point. Single-neuron transcriptome analyses have shown that in adult mice, the expression patterns of proteins upstream of the Reelin signaling pathway, Dab1, VLDLR, and ApoER2, do not differ between excitatory and inhibitory neurons [38]; however, at postnatal day 0, the transcripts of Dab1 were much lower than those of excitatory neurons, but not those of VLDLR and ApoER2 [39] (http://zylkalab.org/datamousecortex, accessed on: 1 June 2020). 

Despite the lack of response of most inhibitory neurons to Reelin, Reelin-rich protein supplementation reduced the density of inhibitory synapses in excitatory neurons without altering the expression of inhibitory synapse-specific proteins in this study. Previous studies have shown that glutamate receptor activity is required for the development of GABAergic synapses in early developing neurons through interactions between excitatory and inhibitory synaptic machinery [22,40,41,42,43]. Given that Reelin stimulates the formation of dendritic spines and excitatory neurotransmission [4,5,6], Reelin may affect the development of inhibitory synapses by modulating glutamatergic activity without direct stimulation of the Akt/S6 signaling pathway in inhibitory neurons. Recent studies have also shown that Reelin reduces the function of presynaptic GABA_B_ receptors in glutamatergic excitatory neurons [44] and that *Reln* haploinsufficiency leads to immature GABAergic synaptic transmission [45]. This study revealed neuron-specific responses of Reelin, as well as a new function of Reelin in GABAergic inhibitory synapse development, which contributes to the cellular mechanisms underlying Reelin-associated neurological disorders.
